# Dietary adaptation for weight loss maintenance at Yale (DAWLY): Protocol and predictions for a randomized controlled trial

**DOI:** 10.3389/fnut.2022.940064

**Published:** 2022-07-28

**Authors:** Xi Fang, Xue Davis, Kyle D. Flack, Chavonn Duncan, Fangyong Li, Marney White, Carlos Grilo, Dana M. Small

**Affiliations:** ^1^Modern Diet and Physiology Research Center, New Haven, CT, United States; ^2^Department of Psychiatry, Yale University School of Medicine, New Haven, CT, United States; ^3^Department of Dietetics and Human Nutrition, College of Agriculture, Foods, and Environment, University of Kentucky, Lexington, KY, United States; ^4^Yale Center for Analytical Sciences, Yale School of Public Health, New Haven, CT, United States; ^5^Department of Social and Behavioral Sciences, Yale School of Public Health, New Haven, CT, United States

**Keywords:** randomized controlled trial, obesity, overweight, oleoylethanolamide, supplementation, dietary intake, Riduzone, weight loss

## Abstract

**Background:**

Current therapies for obesity treatment are effective at producing short-term weight loss, but weight loss maintenance remains a significant challenge. Here we investigate the impact of pre-intervention dietary fat intake on the efficacy of a dietary supplement to support weight loss maintenance. Preclinical work demonstrates that a vagal afferent pathway critical for sensing dietary lipids is blunted by a high-fat diet (HFD), resulting in a reduced preference for a low-fat emulsion and severe blunting of the dopamine (DA) response to the gastric infusion of lipids. Infusion of the gut lipid messenger oleoylethanolamide (OEA), which is also depleted by HFD, immediately reverses this DA blunting and restores preference for the low-fat emulsion. Studies of OEA supplementation for weight loss in humans have had limited success. Given the strong effect of HFD on this pathway, we designed a study to test whether the efficacy of OEA as a weight loss treatment is related to pre-intervention habitual intake of dietary fat.

**Methods/Design:**

We employed a randomized, double-blind, placebo-controlled trial in which 100 adults with overweight/obesity (OW/OB) were randomized to receive either OEA or placebo daily for 16 months. Following a baseline evaluation of diet, metabolic health, adiposity, and brain response to a palatable an energy dense food, participants in both groups underwent a 4-month behavioral weight loss intervention (LEARN^®^) followed by a 1-year maintenance period. The study aims are to (1) determine if pre-intervention dietary fat intake moderates the ability of OEA to improve weight loss and weight loss maintenance after a gold standard behavioral weight loss treatment; (2) identify biomarkers that predict outcome and optimize a stratification strategy; and (3) test a model underlying OEA’s effectiveness.

**Discussion:**

Focusing on interventions that target the gut-brain axis is supported by mounting evidence for the role of gut-brain signaling in food choice and the modulation of this circuit by diet. If successful, this work will provide support for targeting the gut-brain pathway for weight loss maintenance using a precision medicine approach that is easy and inexpensive to implement.

**Clinical Trial Registration:**

[www.ClinicalTrials.gov], identifier [NCT04614233].

## Introduction

Obesity has now reached epidemic proportions in the United States with a prevalence of 42.4% in 2017–2018 (1). This represents an increase of almost 40% from 1999–2000, according to the latest report from Centers for Disease Control and Prevention (1). The increasing prevalence of obesity, and its strong relation to a number of chronic conditions and cancer, escalates the public health significance of this chronic medical problem. Current pharmacological treatments and lifestyle behavioral weight loss interventions for obesity focusing on reducing energy intake and increasing physical activity are effective at producing short-term weight loss (2), but weight loss maintenance remains a significant challenge (2–5). Weight loss reduces morbidity (6) while regain following obesity treatment is associated with increased medical problems (7).

Obesity has been described as a neurobehavioral disorder resulting from a vulnerable brain in an obesogenic environment (8). While central circuits influence body weight through multiple pathways, including neural, immune, and endocrine mechanisms, those underlying nutrient sensing and food choice are increasingly recognized as key targets for behavioral, neural, and pharmacological intervention (9). A vagal afferent pathway that conveys reinforcing signals to the brain was recently identified in rodents. This pathway originates in the upper intestine and projects, *via* the vagus nerve to the nodose ganglion (NG), hindbrain, midbrain, and finally, the dorsal striatum (DS), where it directly regulates dopamine (DA) release to drive reinforcement learning (10). This pathway is sensitive to and activated by dietary lipids but habitual high fat intake can blunt sensitivity. For example, direct infusion of intralipids into the gut of mice maintained on a regular chow diet, but not high fat diet (HFD) results in DA release in the DS (11). This HFD-induced blunting of the DA response is accompanied by a shift in preference away from lower fat emulsions (11). Notably, body weight and metabolism were not altered in the animals maintained on the HFD, indicating that the effect was induced by the diet (11). This aligns with accumulating evidence that a HFD can rapidly alter DA signaling to produce compulsive responses for food (12), devaluation of nutritionally balanced food (13) impulsive (14) and depressive behaviors (15), as well as impaired reinforcement learning (16).

Oleoylethanolamide (OEA) is an endogenous *N-*acylethanolamines (NAE) released from the small intestine in response to ingested fat and functions as an anorexic lipid mediator that promotes satiety (17–19). It exerts anti-inflammatory (20), anti-oxidative (21), and athroprotective effects (22) by enhancing PPARα signaling (23) and modulating lipid profiles (22, 24). HFD dose-dependently reduces the intestinal synthesis of NAE while peripheral but not central administration of OEA persistently reduces food intake in rodents (18, 25). Critically, exogenous administration of OEA rescues the HFD-induced blunted DA response to intralipid infusion in the gut and reverses the blunted preference for low-fat emulsions (11, 26). Consistently, OEA has been demonstrated to aid in weight loss in HFD-induced obesity in rodents (25, 27). However, in humans, studies that do not take diet into account report more modest effects. For example, supplementation improves compliance to energy restricted diets but does not result in greater weight loss when baseline diet is not considered (28, 29). This raises the possibility that OEA might be selectively effective in individuals who regularly consume a HFD diet.

**FIGURE 1:**
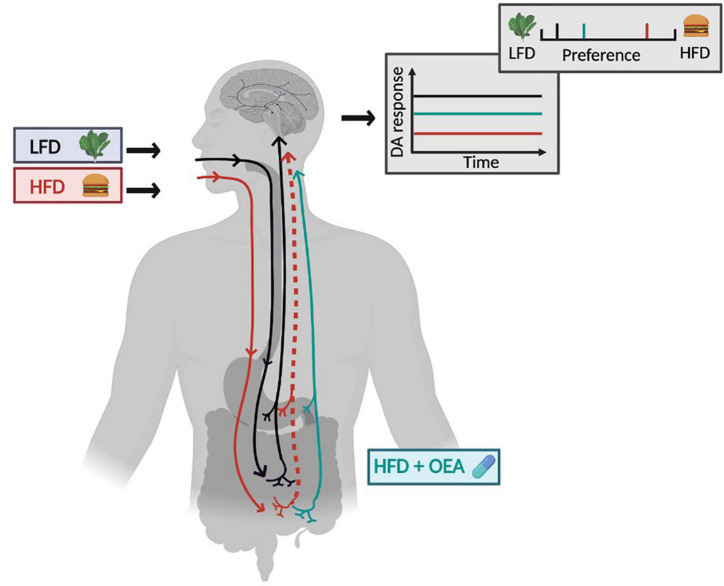
Model of OEA efficacy. The relationship between diet and gut-brain signaling is shown in the context of a low-fat diet (LFD, gray box with lettuce) and a high-fat diet (HFD, red box with hamburger). Following habitual consumption HFD the vagal afferent pathway that ascends from intestine to the brain is blunted (dotted line) and this is associated with a blunted dopamine (DA) response to nutrients (red line on graph) compared to DA response to nutrients in LFD consumers (black line on graph). DA responses are associated with fat concentration preference, depicted in the preference scale above the graph. Preferences are shifted to the right in the context of a HFD. Supplementation with OEA in the HFD consumers is shown in cyan. Supplementation rescues the blunted vagal afferent pathway leading to a recovered DA response to nutrients (cyan line in graph) and a shift in preference toward low fat food. HFD, high-fat diet; LFD, low-fat diet; DA, dopamine; OEA, oleoylethanolamide. Figure created in Biorender.

DAWLY (Dietary Adaptation for Weight Loss maintenance at Yale) is a randomized controlled clinical trial designed to evaluate the efficacy of supplementation with OEA to improve weight loss maintenance in HFD consumers. The aims are to: (1) determine if HFD moderates the ability of OEA to improve weight loss maintenance after a gold-standard behavioral weight loss intervention (LEARN^®^) in individuals with overweight/obesity (OW/OB); (2) examine if dietary fat and sugar intake questionnaire (DFS) is associated with measures of saturated fat intake, optimize a clinically useful stratification strategy; and (3) assess a model of OEA effectiveness. We predict that fat intake will strongly moderates the ability of OEA to produce clinically significant weight loss maintenance and that the DFS will prove to be a valid marker of fat intake and therefore OEA effectiveness. Finally, we will test a model of treatment efficacy (Figure 1). Specifically, we propose that weight loss outcome is associated with shifts in fat preference and intake and that these effects are mediated by changes in striatal and/or insular response to milkshake in the OEA but not the placebo group. The striatum and insula were chosen as regions of interest based on animal data presented above and neuroimaging studies reporting that response in these regions changes with dietary manipulations (30–32). We will further test whether HFD is associated with performance on reinforcement and cognitive measures or changes in energy expenditure or substrate utilization. If so, we will test whether these associations and their reversal by OEA contribute to our outcomes (Table 1). Findings from this study have the potential to support a novel gut-brain target for weight loss maintenance in combination with a precision medicine approach to behavioral weight loss plus supplementation that is easy and inexpensive to implement. In this paper, we describe the study design, protocol, and analysis plan.

**TABLE 1:** Primary and secondary outcome measures.

Primary outcome measures:	
1.	Percent change in body weight [Time Frame: Baseline, 4 month, 8 month, and 16 months]: Weight in kg will be used to measure the percent change in body weight.
2.	Change in absolute body weight [Time Frame: Baseline, 4 month, 8 month, and 16 months]: Weight in kg will be used to measure absolute body weight
3.	Change in waist circumference [Time Frame: Baseline, 4 month, 8 month, 16 month]: Waist circumference to the nearest 0.1 cm
4.	Change in hip circumference [Time Frame: Baseline, 4 month, 8 month, and 16 months]: Hip circumference to the nearest 0.1 cm
5.	Change in thigh circumference [Time Frame: Baseline, 4 month, 8 month, and 16 months]: Thigh circumference to the nearest 0.1 cm
6.	Change in body fat percent [Time Frame: Baseline, 4 month, 8 month, and 16 months]: Percentage of body fat measured with bioimpedance analysis
7.	Change in visceral adipose tissue [Time Frame: Baseline, 4 month, 8 month, 16 month]: Visceral adipose tissue in liters measured with bioimpedance analysis
8.	Change in saturated fat intake as measured by the DFS [Time Frame: Baseline, 4 month, 8 month, and 16 months]: Dietary fat intake will be measured monthly by diet fat and sugar intake questionnaire (DFS) and for 16 months starting from baseline. Higher scores indicate a higher frequency of saturated fat intake.
9.	Change in saturated fat intake as measured by the ASA24 [Time Frame: Baseline, 4 month, 8 month, and 16 months]: Saturated fat intake will be measured monthly by the automated self-administered 24-h recall (ASA24) for 16 months starting from baseline saturated fat intake will be reported in grams.
10.	Change in total saturated fat intake as measured by 3-day food diaries [Time Frame: Baseline, 4 month, 8 month, and 16 months]: Saturated fat intake will be measured monthly by 3-day food diaries for 16 months starting from baseline. The 3-day food diaries will be processed with the Nutrition Data System for Research (NDSR). Saturated fat intake will be reported in grams.
11.	Change in total fat intake as measured by 3-day food diaries [Time Frame: Baseline, 4 month, 8 month, and 16 months]: Fat intake will be measured monthly by 3-day food diaries for 16 months starting from baseline. The 3-day food diaries will be processed with the Nutrition Data System for Research (NDSR). Total fat intake will be reported in grams.
12.	Change in total saturated fat intake as measured by C-reactive protein [Time Frame: Baseline, 8 month]: Saturated fat intake will be measured by plasma C-reactive protein in mg/L.
13.	Change in brain response to milkshake [Time Frame: baseline, 8 month]: The Blood-oxygen-level-dependent response (BOLD) in the dorsal striatum to the taste of milkshake and tasteless stimuli will be measured using a 3T fMRI scanner at baseline for all participants and at month 8 for participants in the intervention group
14.	Change in fat perception with Visual Analog Scale [Time Frame: Baseline, 4 month, 8 month, and 16 months]: Fat perception will be measured with the Visual Analog Scale. Participants will be asked to sample Jello’s and puddings of differing fat concentrations and rate their perceptual attributes at baseline, month 4, month 8, and month 16 The Visual Analog Scale (VAS) will be used to assess perceptual attributes such as oiliness, fattiness, and creaminess, while accounting for hunger, fullness, thirst, and wanting. The VAS is a horizontal line anchored by ‘not at all’ at one end and ‘extremely’ at the other. The scale value of ‘not at all’ will be 0% and the scale value of ‘extremely’ will be 100%. Higher values indicate that a subject perceives these attributes as more intense.
15.	Change in fat perception with General Labeled Magnitude Scale [Time Frame: Baseline, 4 month, 8 month, and 16 months]: Fat perception will be measured with the General Labeled Magnitude Scale (GLMS). Participants will be asked to sample Jello’s and puddings of differing fat concentrations and rate their perceptual attributes at baseline, month 4, month 8, and month 16 The GLMS will assess intensity perception. The GLMS is a vertical line with quasi-logarithmic spaced labels that start at the bottom ‘barely detectable’ to ‘strongest imaginable’ at the top.
16.	Change in fat preference with Labeled Hedonic Scale [Time Frame: Baseline, 4 month, 8 month, and 16 months]: Fat preference will be measured. Participants will be asked to sample Jello’s and puddings of differing fat concentrations and rate their hedonic attributes at baseline, month 4, month 8, and month 16 The Labeled Hedonic Scale (LHS) will assess liking. The LHS is a vertical line with quasi-logarithmic spaced labels that start at the bottom with ‘most imaginable dislike’ and go to ‘most imaginable like’ at the top, with the label ‘neutral’ in the middle. The scale value of ‘most imaginable dislike’ will be −100; the scale value of ‘neutral’ will be 0; the scale value of ‘most imaginable like’ will be 100. Higher values indicate greater liking of the sample.
17.	Change in sugar perception with General Labeled Magnitude Scale [Time Frame: Baseline, 4 month, 8 month, and 16 months]: Sugar perception will be measured with the General Labeled Magnitude Scale. Participants will be asked to sample Jello’s and puddings of differing sugar concentrations and rate their perceptual and attributes at baseline, month 4, month 8, and month 16 The GLMS will assess intensity perception. The GLMS is a vertical line with quasi-logarithmic spaced labels that start at the bottom ‘barely detectable’ to ‘strongest imaginable’ at the top.
18.	Change in sugar preference with Labeled Hedonic Scale [Time Frame: Baseline, 4 month, 8 month, and 16 months]: Sugar perception will be measured with the Labeled Hedonic Scale. Participants will be asked to sample Jello’s and puddings of differing sugar concentrations and rate their hedonic attributes at baseline, month 4, month 8, and month 16 The LHS will assess liking. The LHS is a vertical line with quasi-logarithmic spaced labels that start at the bottom with ‘most imaginable dislike’ and go to ‘most imaginable like’ at the top, with the label ‘neutral’ in the middle
19.	Change in fat concentration preference measured by the Monell forced choice test [Time Frame: Baseline, 4 month, 8 month, and 16 months]: Fat concentration preference will be measured by the Monell forced-choice test. Participants will be asked to sample Jello’s and puddings of differing fat concentrations at baseline, month 4, month 8, and month 16. Subjects will be presented with pairs of puddings of varying fat concentrations in a two-series test. Subjects will taste two puddings of different fat concentrations and indicate their preferred pudding. The following pairs of puddings will be presented based on the subject’s previous choice. This will continue until selection of the same fat concentration twice relative to both a lower and a higher concentration, or until the lowest or highest concentration is chosen twice consecutively.
20.	Change in sweet concentration preference measured by the Monell forced choice test [Time Frame: Baseline, 4 month, 8 month, and 16 months]: Sweet concentration preference will be measured by the Monell forced-choice test. Participants will be asked to sample Jello’s and puddings of differing sugar concentrations at baseline, month 4, month 8, and month 16. Subjects will be presented with pairs of puddings/Jello’s of varying sugar concentrations in a two-series test. Subjects will taste two puddings/Jello’s of different sugar concentrations and indicate their preference. The following pairs of puddings/Jello’s will be presented based on the subject’s previous choice. This will continue until selection of the same sugar concentration twice relative to both a lower and a higher concentration, or until the lowest or highest concentration is chosen twice consecutively.
21.	Change in food reinforcement with RED-13 [Time Frame: Baseline and 8 month]: The relative reinforcing value of foods will be measured using the Reward-Related Eating (RED-13) Questionnaire. This questionnaire results in a score from 0 as the lowest measure of reward-based eating drive and 36 as the highest.
22.	Change in food reinforcement with Becker DeGroot Markov Auction Task [Time Frame: Baseline and 8 month]: The relative reinforcing value of foods will be measured using a computerized auction task at baseline and month 8. The relative reinforcing value of foods will be measured with a modified version of the Becker DeGroot Markov Auction task. The outcome measure is willingness to pay, such that higher willingness to pay indicates higher relative reinforcing value of foods.
23.	Change in cognition with Kirby Delay Discounting [Time Frame: Baseline and 8 month]: A neuropsychological test battery will be performed to assess obesity or dopamine-related differences in cognition at baseline and month 8. The neuropsychological test battery will include Kirby Delay Discounting, which measures temporal discounting (tendency for people to prefer small, immediate, monetary rewards over larger, delayed rewards). The steepness of the discounting curve represents the tendency for temporal discounting, such that a more steeply declining curve indicates a tendency to devalue rewards as they become more temporally remote.
24.	Change in cognition with the Relational Task [Time Frame: Baseline and 8 month]: A neuropsychological test battery will be performed to assess obesity or dopamine-related differences in cognition at baseline and month 8. The neuropsychological test battery will include the Relational Task which measures visual relational processing. The number of correct responses in the relational condition is the outcome measurement, such that more correct responses indicates higher visual relational processing.
25.	Change in cognition with Penn Progressive Matrices Test [Time Frame: Baseline and 8 month]: A neuropsychological test battery will be performed to assess obesity or dopamine-related differences in cognition at baseline and month 8. The neuropsychological test battery will include the Penn Progressive Matrices Test, which measures fluid intelligence. The number of correct responses is the outcome measurement, such that more correct responses indicates higher fluid intelligence.
26.	Change in cognition with Oral Reading Recognition Test [Time Frame: Baseline and 8 month] A neuropsychological test battery will be performed to assess obesity or dopamine-related differences in cognition at baseline and month 8. The neuropsychological test battery will include the Oral Reading Recognition Test, which measures language decoding and reading. The score based on accuracy is the outcome measure, such that a higher score indicates higher language decoding and reading ability
27.	Change in cognition with Variable Short Penn Line Orientation Test [Time Frame: Baseline and 8 month]: A neuropsychological test battery will be performed to assess obesity or dopamine-related differences in cognition at baseline and month 8. The neuropsychological test battery will include the Variable Short Penn Line Orientation Test, which measures visuospatial processing. The number of correct responses is the outcome measure, such that a higher number of correct responses indicates higher visuospatial processing ability.
28.	Change in cognition with Matrix Reasoning Task (Core NMOB) [Time Frame: Baseline and 8 month]: A neuropsychological test battery will be performed to assess obesity or dopamine-related differences in cognition at baseline and month 8. The neuropsychological test battery will include the Core Neuropsychological Measures for Obesity and Diabetes (Core NMOB). Core NMOB includes the Matrix Reasoning task, which reflects general cognitive ability or non-verbal reasoning ability. Accuracy across 35 trials is the outcome measure, such that higher accuracy indicates higher general cognitive ability or non-verbal reasoning ability.
29.	Change in cognition with Digit Symbol Substitution (Core NMOB) [Time Frame: Baseline and 8 month]: A neuropsychological test battery will be performed to assess obesity or dopamine-related differences in cognition at baseline and month 8. The neuropsychological test battery will include the Core Neuropsychological Measures for Obesity and Diabetes (Core NMOB). Core NMOB includes the Digit Symbol Substitution task, which reflects processing speed. The number of correctly matched symbols, within the administration time (90 s) is the outcome measure, such that higher number of correctly matched symbols indicates higher processing speed.
30.	Change in cognition with Go/No-Go Task (Core NMOB) [Time Frame: Baseline, 4 month, 8 month, and 16 months]: A neuropsychological test battery will be performed to assess obesity or dopamine-related differences in cognition at baseline, month 4, month 8, and month 16. The neuropsychological test battery will include the Core Neuropsychological Measures for Obesity and Diabetes (Core NMOB). Core NMOB includes the Go/No-Go task, which reflects response inhibition. The sensitivity index (d’) and commission error rate is the outcome measure, such that higher d’ and lower commission error rates indicate higher response inhibition.
31.	Change in cognition with Penn Word Memory Test [Time Frame: Baseline and 8 month]: A neuropsychological test battery will be performed to assess obesity or dopamine-related differences in cognition at baseline and month 8. The neuropsychological test battery will include the Penn Word Memory Test, which measures verbal episodic memory. The number of correct responses is the outcome measure, such that a higher number of correct responses indicates higher verbal episodic memory.
32.	Change in cognition with Dimensional Change Card Sorting (Core NMOB) [Time Frame: Baseline and 8 month]: A neuropsychological test battery will be performed to assess obesity or dopamine-related differences in cognition at baseline and month 8. The neuropsychological test battery will include the Core Neuropsychological Measures for Obesity and Diabetes (Core NMOB). Core NMOB includes the Dimensional Change Card Sorting task, which reflects cognitive flexibility and task-switching ability. The accuracy score is the outcome measure, such that higher accuracy indicates higher cognitive flexibility and task-switching ability.
33.	Change in cognition with Picture Sequence Memory (Core NMOB) [Time Frame: Baseline and 8 month]: A neuropsychological test battery will be performed to assess obesity or dopamine-related differences in cognition at baseline and month 8. The neuropsychological test battery will include the Core Neuropsychological Measures for Obesity and Diabetes (Core NMOB). Core NMOB includes the Picture Sequence Memory task, which reflects learning and memory. The accuracy score is the outcome measure, such that higher accuracy indicates higher learning and memory abilities.
34.	Change in cognition with Stockings of Cambridge (SOC) test (CANTAB) [Time Frame: Baseline and 8 month]: A neuropsychological test battery will be performed to assess obesity or dopamine-related differences in cognition at baseline and month 8. The neuropsychological test battery will include the Cambridge Neuropsychological Test Automated Battery (CANTAB). The CANTAB includes the Stockings of Cambridge test, which reflects spatial planning. Outcome measures assess the problem difficulty level reached, mean moves used and thinking time are the outcome measures. Scores will be compared to normative data from age and sex-matched peers
35.	Change in cognition with Intra-Extra Dimensional Set Shift test (CANTAB) [Time Frame: Baseline and 8 month]: A neuropsychological test battery will be performed to assess obesity or dopamine-related differences in cognition at baseline and month 8. The neuropsychological test battery will include the Cambridge Neuropsychological Test Automated Battery (CANTAB). The CANTAB includes the Intra-Extra Dimensional Set Shift test, which reflects rule acquisition and reversal. Outcome measures assess the number of errors made, the number of trials completed, the number of stages completed and latency. Scores will be compared to normative data from age and sex-matched peers.
36.	Change in cognition with delayed non-matching to sample test (CANTAB) [Time Frame: Baseline and 8 month]: A neuropsychological test battery will be performed to assess obesity or dopamine-related differences in cognition at baseline and month 8. The neuropsychological test battery will include the Cambridge Neuropsychological Test Automated Battery (CANTAB). The CANTAB includes the delayed non-matching to sample test, which reflects visuospatial memory. Outcome measures include latency (the participant’s speed of response), the number of correct patterns selected and a statistical measure giving the probability of an error after a correct or incorrect response. Scores will be compared to normative data from age and sex-matched peers
37.	Change in cognition with Paired Associates Learning task (CANTAB) [Time Frame: Baseline and 8 month]: A neuropsychological test battery will be performed to assess obesity or dopamine-related differences in cognition at baseline and month 8. The neuropsychological test battery will include the Cambridge Neuropsychological Test Automated Battery (CANTAB). The CANTAB includes the Paired Associates Learning task, which assesses episodic memory and new learning. Outcome measures include the errors made by the participant, the number of trials required to locate the pattern(s) correctly, memory scores and stages completed. Scores will be compared to normative data from age and sex-matched peers.
38.	Change in cognition with Probabilistic-Feedback Reward Task [Time Frame: Baseline, 4 month, 8 month, and 16 months]: A neuropsychological test battery will be performed to assess obesity or dopamine-related differences in cognition at baseline, month 4, month 8, and month 16. The neuropsychological test battery will include the Probabilistic-Feedback Reward Task, which assesses the ability to learn from positive and negative outcome. Outcome measures include the number of times a symbol associated with positive feedback is chosen and the number of times a symbol associated with negative feedback is avoided. Scores will be compared to normative data from age and sex-matched peers.
39.	Change in resting energy expenditure [Time Frame: Baseline and 8 month]: Indirect Calorimetry (IC) will be performed to measure fasting resting energy expenditure at baseline and month 8.
40.	Change in respiratory exchange ratio [Time Frame: Baseline and 8 month]: Indirect Calorimetry (IC) will be performed to measure fasting respiratory exchange ratio at baseline and month 8
**Secondary outcome measures**	
1.	Change in Healthy Eating Index (HEI) [Time Frame: Baseline, 4 month, 8 month, 16 month]: The overall Healthy Eating Index 2015 (HEI-2015) score will be calculated for each participant from 3-day food diaries processed through NDSR. This score is made up of 13 components that reflect recommendations in the 2015–2020 Dietary Guidelines for Americans. The maximum score for the HEI is 100, where points are awarded based on adequate intakes of total fruit, whole fruits, total vegetables, greens and beans, whole grains, dairy, protein foods, seafood and plant proteins, and unsaturated fatty acids. Points are also awarded for moderate intakes of refined grains, sodium, added sugars and saturated fats.
2.	Change in 3-day food diary total solid fat intake [Time Frame: Baseline, 4 month, 8 month, 16 month]: Solid fat intake will be measured monthly by 3-day food diaries for 16 months starting from baseline. The 3-day food diaries will be processed with the Nutrition Data System for Research (NDSR) and solid fat intake will be reported in grams.
3.	Change in saturated fat intake measured by plasma cholesterol [Time Frame: Baseline, 8 month]: Plasma cholesterol LDL/HDL will be measured at baseline and month 8.
4.	Change in saturated fat intake measured by plasma triglycerides [Time Frame: Baseline, 8 month]: Plasma triglycerides will be measured at baseline and month 8

## Methods and analysis

### Study design

#### Design and setting

**FIGURE 2:**
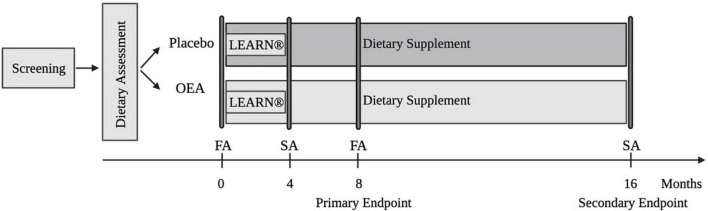
Study design. OEA, oleoylethanolamide; FA, full assessment; SA, short assessment.

The design of DAWLY, a single-center, longitudinal, randomized, double-blinded, placebo-controlled trial is depicted in Figure 2. The trial involves 100 adults with overweight/obesity (OW/OB) randomized into two treatment groups (50 OEA, 50 placebo). We will evaluate the long-term effects of daily OEA supplementation over the 16 months of treatment before and after a 4-month behavioral weight loss intervention (LEARN^®^). We will]determine if dietary fat intake moderates the ability of OEA to improve weight loss and weight loss maintenance after LEARN^®^ (Aim 1); identify biomarkers that predict outcome, optimize a stratification strategy (Aim 2); and test a model underlying OEA’s effectiveness (Aim 3).

#### Recruitment

We plan to screen 175 OW/OB individuals to secure 120 participants for an initial 3-day food diary assessment and enroll 100 subjects between April 2021 and April 2024. Due to study delays and lower recruitment rates caused by the pandemic, we may extend our window of enrollment beyond April 2024. At the time of writing, 14 participants have been enrolled, with no adverse side-effects and ongoing maintenance of blinding. We will continue to recruit until 100 participants complete the baseline full assessment (FA) and are subsequently assigned randomly to either the OEA or Placebo aim to begin the 16-month trial. Participants are recruited using advertisements on flyers, Facebook, Instagram, city transit (e.g., posters on buses), billboards, radio, Yale Center for Clinical Investigation, and the laboratory subject database.

#### Eligibility criteria

The inclusion and exclusion criteria are listed in Table 2. A number of strategies are in place to maximize retention and minimize attrition, including the flexibility with visit scheduling, 24/7 accessibility of the researchers, automated reminders, instant contact with missing sessions, transportation reimbursement, and financial incentives for completing the visits and completion bonuses at endpoints.

**TABLE 2:** Inclusion and exclusion criteria.

Inclusion criteria:
Ages 18–55
Right-handed with a score of ≥ + 50 on the modified Edinburgh handedness scale
English-speaking
BMI > 25.0
Comfortable with the fMRI procedures during the mock scanning session
Rate milkshake as at least mildly liked
Interested in weight loss
**Exclusion criteria:**
Serious or unstable medical illness (e.g., cancer)
Past or current history of alcoholism or consistent drug use
Current major psychiatric illnesses including eating disorders
Medications that affect alertness (e.g., barbiturates, benzodiazepines, chloral hydrate, haloperidol, lithium, carbamazepine, phenytoin, etc.)
History of major head trauma with loss of consciousness
Ongoing pregnancy
History of metalworking, injury with shrapnel or metal slivers, or major surgery
History of pacemaker or neurostimulator implantation
Known taste or smell dysfunction
A diagnosis of diabetes
Food allergy to dairy products
Tobacco use
Supplements that will influence fat metabolism
Vegan

#### Randomization

A randomization list with 1:1 ratio, created by a lab member not affiliated with the study, is used to assign eligible subjects into the OEA group or the Placebo control group while balancing age, BMI, and years of education between the two arms.

### Experimental measures

#### Dietary assessment

The National Cancer Institute (NCI) Dietary Assessment Primer recommends combining food frequency questionnaire (FFQ) with dietary recalls to exploit the advantages and minimize the weakness of each approach (33). Therefore, the DFS (34) and the Automated Self-Administered 24-h (ASA24) dietary assessment is delivered once per month over the whole trial (16 months). In addition, weighted food records (3-Day Food Record) are used during intake, after the LEARN^®^ program, just prior to the primary endpoint, and just prior to the secondary endpoint to assess specific quantities of foods and deduce total energy and macronutrient intake.

The DFS comprises 26 questions about the frequency of foods and beverages consumed over the past 12-months (34). The DFS has three subscales to estimate consumption of food items that are high in saturated fat and low in sugar, food items that are high in free sugars and low in saturated fat, and food items that are high in both saturated fat and free sugars. A total score reflecting dietary intake of saturated fat and free sugar is derived by summing the scores from each subscale.

The ASA24 is a web-based tool that enables multiple, automatically coded, and self-administered 24-h dietary recalls. Participants are asked for the time and content of the meal as well as information on where and with whom the meal is consumed. The system guides the participant through the multiple passes of dietary intake using prompts and a list of foods and beverages from the USDA Food and Nutrient Database for Dietary Studies. Multiple images are shown to help respondents estimate portion size. If food items are not found in the database, they can be entered manually.

Weighed food records are commonly viewed as the “gold standard” for self-reported dietary intake assessment and have been used as the reference method in validation studies offering high validity and precision (35). Our implementation includes two weekdays and one weekend day at baseline, post-LEARN^®^ and twice during the maintenance phase. Participants are provided a food scale to weigh all food and beverages consumed during each 3-day period. Participants are also provided a food journal to list other details of their dietary intake, including the time and place they consumed the food/drink, where the food came from, how it was prepared, and any additional toppings or condiments. The 3-day food diary will be analyzed using the Nutrition Data System (NDS) for Research by a registered dietitian (Dr. Flack).

#### Anthropometric assessment

Waist, hip, and thigh circumferences are assessed with a tape measure, and height is measured to the nearest 0.1 cm using a stadiometer (Itin Scale Co., Inc., Germany). Bodyweight, body fat percentage, and visceral adipose tissue are calculated using a Seca mBCA 514 medical Body Composition Analyzer (Itin Scale Co., Inc., Germany) at screening, baseline, months four, eight and 16.

#### Neuroimaging

**FIGURE 3:**
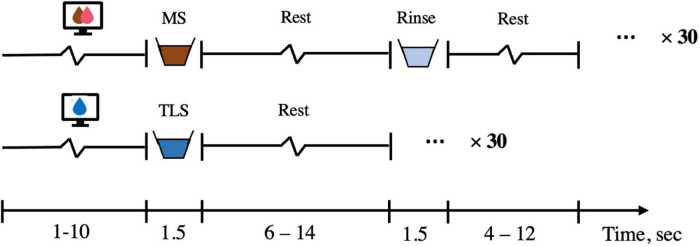
fMRI solution presentation. The solution run is 16-min long and consisted of a 1–10 s visual cue (average 3 s), a 1.5 s delivery of either chocolate/strawberry milkshake or tasteless solution, followed by a 6–14 s rest period (average 8 s) during which the subject could swallow. If milkshake is delivered, a 1.5 s rinse of deionized water then occurred and was followed by a 4–12 s rest period (average 6 s). There are 30 repetitions of milkshake and 30 repetitions of tasteless run. MS, milkshake; TLS, tasteless.

All fMRI scans are scheduled between 8:00 AM and 12:00 PM. Participants are instructed to arrive neither full nor hungry, and adherence to these instructions is confirmed with subjective ratings of participant internal states of hunger, fullness, and thirst using visual analog scales (VAS). Structural and functional imaging data is collected on a Siemens Prisma scanner at the Yale Magnetic Resonance Research Center, using the following scan parameters: high resolution T1-weighted structural scan repetition time (TR) = 1,900 ms, echo time (TE) = 2.52 ms, slice thickness = 1 mm, flip angle = 9, field of view (FOV) = 250 × 250, matrix = 256 × 256, 176 slices; functional milkshake run TR = 1,500 ms, TE = 34 ms, slice thickness = 2 mm, flip angle = 70, FOV = 192 × 192, matrix = 96 × 96, 72 interleaved slices. The fMRI scans are consist of one 16-min run that consists of 60 cued presentations of milkshake or tasteless solutions delivered in pseudorandomized order (Figure 3). These solutions are delivered in quantities of 0.5 mL over a 1.5 s period followed by 8 s to swallow using a specially designed gustometer. After each milkshake delivery, the participant receives a rinse consisting of 0.5 mL of deionized water over a period of 1.5 s and is given 6 s to swallow.

#### Neuropsychological assessments

We use the brief neuropsychological battery for obesity (BNBO) to assess general cognitive function associated with obesity (36). The BNBO includes the Kirby Delay Discounting, the Relational Task, the Penn Progressive Matrices Test, the Oral Reading Recognition Test, the Variable Short Penn Line Orientation test, and the Penn Word Memory Test. All tests are well-validated and freely available. The NIH-recommended Core Neuropsychological Measures for Obesity and Diabetes Trials (Core-NMOB) is also used (37). The battery includes the brain health index (BHI), which includes Matrix Reasoning and Digit Symbol Substitution and is sensitive to individual differences in overall cognition. The BHI also provides standard measures that can be used to interpret performance on other measures.

To assess executive functions and decision making the Go/No-Go task, the Dimensional Change Card Sorting, the Picture Sequence Memory, and the Kirby Delay Discounting task are administered. Given the strong evidence that HFD can alter DA-function and blunt the reinforcing vagal afferent pathway, which is reversed by OEA administration, we administer a series of DA-dependent and DA-independent tasks (38, 39). The spatial working memory task (SWM) is DA-dependent and the delayed non-matching to sample test (DMS) is DA-independent (40). In particular, the SWM has been associated with the structural integrity of the dorsal striatum, the integrity of DA-dependent frontostriatal circuits, and is altered by pharmacological manipulation of DA signaling (41, 42). In contrast, pharmacological manipulation of DA signaling has no influence on the performance of the DMS (40). Executive function is measured with the Stockings of Cambridge (SOC) test of spatial planning and the Intra-Extra Dimensional Set Shift test of rule acquisition and reversal. The SOC is DA-dependent and the Set Shift task is sensitive to pharmacological manipulation of noradrenergic, but not DAergic frontostriatal circuit function (41). DA-dependent reinforcement learning (positive and negative outcome learning) is measured with the Probabilistic-Feedback Reward Task (PFRT), which has multiple versions allowing repeated testing and evidence that practice effects do not affect relative positive vs. negative feedback learning (43). Finally, visual learning and memory is also assessed with the Paired Associates Learning task, which is DA-independent (41).

#### Metabolic measurement

Indirect calorimetry (IC) is used to measure resting energy expenditure (REE) and the respiratory exchange ratio (RER) (44). The assessment is conducted between 8:00 and 9:00 AM following a 10–12 h overnight fast. The participant is asked to abstain from alcohol, caffeine, and moderate exercise for at least 2 h prior, and vigorous resistance exercise for at least 14 h prior to measurement. The participant rests supine for 10–20 min followed by 30 min of data collection to obtain steady-state RER and REE. Data is analyzed based on Compher et al.’s best practice methods (44). The first 5 min are discarded and the 5 min segment with the smallest fluctuations of CO_2_ and O_2_ values with a coefficient of variation ≤ 10% is identified. RER and REE are averaged across the 5-min.

#### Food reinforcement testing

We have recently developed a modified version of the Becker DeGroot Markov Auction task (45) that allows us to test the relative reinforcing value of snack foods that are matched for energy content, liking, and familiarity, but vary in macronutrient content. In particular, the task has three categories of snacks: (1) High in fat and low in carbohydrate (FAT), (2) High in carbohydrate and low in fat (CARB), and (3) High in both fat and carbohydrate (COMBO). We have used this task during fMRI scanning and have shown that people bid more for COMBO foods than foods with FAT or CARB even though energy content, liking and familiarity does not vary across the categories (46). Further, this effect is associated with supra-additive responses in the DS (47). Here we use this task to determine if the reinforcing value of fat predicts outcome or is affected by HFD and OEA. In addition, participants complete the Reward-Related Eating (RED-13) Questionnaire (48), which is a psychometrically sound scale that captures lack of control over eating, lack of satiety and preoccupation with food.

#### Perceptual testing

During the baseline and month eight follow-up assessments, fat and sweet concentration preference and perception are measured. Two sets of stimuli that vary in either fat content (pudding) or sucrose concentration (Jell-O^®^) are rated according to perceptual and hedonic attributes (pudding samples prepared with 0%, 1.6%, 3.1%, and 6.9% fat weight by weight (w/w) (49) and set of four Kool-Aid-based Jell-O^®^ s with 0, 0.1, 0.56 and 1.0 M sucrose). Overall stimulus intensity and sweetness are rated using the general labeled magnitude scale (50), fattiness, creaminess, and wanting are rated using a visual analog scale, and liking is rated using the labeled hedonic scale (51). Pudding and Jell-O^®^ stimulus sets are presented by spoon in separate blocks and each concentration repeated 3X in a randomized order. Visual analog scales are used to rate feelings of hunger, fullness, and thirst before and after completing the test.

#### Laboratory measures

Blood is collected at baseline and month eight follow-up for measurement of plasma *N-*acyl phosphatidylethanolamine-specific phospholipase D (NAPE-PLD), cholesterol low-density lipoprotein (LDL)/high-density lipoprotein (HDL), triglycerides (TG), C-reactive protein (CRP), interleukin-6 (IL-6), nucleus factor kappa B (NFkB), tumor necrosis factor alpha (TNF-α), insulin, glucose, and hemoglobin A1C (HbA1c) using commercially-available kits. Plasma levels of OEA are analyzed by Cayman Chemical^1^.

### Protocol

The DAWLY study protocol, depicted in Figure 1, involves nine in-person visits and twelve virtual visits over a 16-month period. During the screening session, the full study procedure is explained and consent is obtained. The Beck Depression Inventory II (BDI) is used to index depressive symptoms (52). Physical activity over the last 7 days is assessed using the International Physical Activity Questionnaire (iPAQ) (53). The baseline and the follow-up full assessments at month eight each comprise three sessions. The first includes neuroimaging, the second metabolic measurements and blood collection, and the third neuropsychological assessment, food reinforcement testing, and perceptual testing. There are also two short assessments, one at the end of the fourth month and one at the end of the 16th month. The short assessments include dietary assessment, anthropometric assessment, perceptual testing, and an abbreviated version of neuropsychological assessment including the Go/No-Go task and PFRT.

During the entire study, dietary intake is measured by DFS and ASA24 every month, and the 3-day food diary is recorded at baseline, and at months four, eight and 16.

#### OEA supplementation

Participants consume OEA supplements (Riduzone^®^)/ placebo daily. Each capsule of Riduzone^®^ contains 200 mg of 90% OEA (180 mg OEA per capsule) and dosing is two servings daily (two capsules/serving = four capsules per day), 15–30 min before a meal, as recommended by Nutriforward LLC. A similar dose has been successfully used in prior clinical trials (19, 20, 54). Additionally, in rodents, oral administration of OEA has been shown to be safe up to 200 mg/kg (55, 56) -the equivalent of 18,144 mg of OEA for a 200 lb (90 kg) human. The OEA content of the pills is assessed annually using mass spectroscopy at the Yale Keck facility.

#### Behavioral weight loss intervention

The LEARN^®^ Program for Weight Management (57) is administered in 12 one-on-one, 45-min sessions (eight weekly, four bi-weekly) by trained behavioral weight loss clinicians, over a 16-week period following the manualized LEARN^®^ Program. LEARN^®^ is an acronym for lifestyle, exercise, attitudes, relationships, and nutrition. This program, which is used widely in treatment trials for obesity (58) and for patients with eating/weight problems (59) uses behavioral methods (goal setting, self-monitoring, problem-solving) to assist patients in making gradual lifestyle changes with goals of sustainable moderate caloric restriction and increased physical activity geared to produce gradual weight losses of about one to two pounds weekly. During these sessions, participants are instructed in food choices and quantities to aim for goals of 1,200–1,500 kcal/day for women and 1,500–1,700 kcal/day for men. The program teaches how to use the behavioral methods to monitor daily food intake and physical activity and to identify ways to make gradual and sustainable changes. The program also teaches behavioral skills to help overcome barriers and challenges to making and sustaining the changes.

#### Compliance and safety monitoring

Study compliance is assessed at each visit through questioning by the clinician and reminders to be sure to take their pills. Participants also fill out virtual questionnaires weekly in which they are asked to record their supplement consumption and indicate possible side effects (Table 3). If a participant does not fill out the e-questionnaire, or if they indicate that they forgot to take the supplement or experienced side effects, a follow-up phone call is made to determine the cause of the failed compliance and to identify solutions. In addition, participants are asked to bring in their remaining pills at the completion of the LEARN^®^, program, as well as at the follow-up full assessment at month eight and the final short assessment at month 16. Pills are collected and compared to the expected usage.

**TABLE 3:** Weekly supplement record.

Over the last week:
Have you taken the provided supplement EVERY DAY?
If no, how many days did you forget?
Have you taken OTHER dietary supplements (besides the one we gave you)? If yes: What were they? What dosage did you take? How often did you take them?
Have you experienced any side effects from the supplements in the past week (e.g., Nausea, vomit, diarrhea, digestive distress, headache, etc.)?
If yes: Please describe your symptoms:

#### Data monitoring and management

Data are collected and maintained using the Research Electronic Data Capture (REDCap) web-based application for building and managing online surveys, questionnaires, and databases. REDCap is also used to monitor submission of the measures and to notify study personnel when measures are not submitted so that the participant can be contacted and reminded. Paper study records are also collected during study visits and secured in locked filing cabinets in the modern diet and physiology research center (MDPRC) office, which is accessible only *via* ID card swipe entry. Digital study records are stored on secure lab servers maintained by Yale University off-site that are backed up weekly with access limited to the PI and research team members. Confidentiality of all information in the study is maintained by identifying subjects by code numbers. No subjects are identified by name in any of the published literature and only by code in data storage areas, to which access is limited to study personnel. The Yale Human Investigation Committee may inspect all study records.

### Statistical considerations

Linear mixed-effect models repeated measure analyses will be used to test the longitudinal effects of body weight, anthropometry, dietary intake, fat/sugar preference, neuropsychological function, and metabolism following dietary supplementation and the LEARN^®^ intervention. The primary endpoint is the follow-up full assessment at month eight, which is 4 months after the completion of the behavioral weight loss trial. We will compare each of the outcome measurements among OEA and Placebo groups across all five time points. Neuroimaging data will be processed using a custom pipeline for taste studies as described below, which uses a combination of tools from a variety of neuroimaging-specific software packages to optimize implementation of each state of preprocessing. Striatal and insular response to milkshake is analyzed using SPM12 (Statistical Parametric Mapping, Wellcome Department of Imaging Neuroscience, London, United Kingdom) in MATLAB R2016b (Mathworks, Inc., Sherborn, MA, United States) where milkshake (MS) and tasteless (Tlss) presentation are modeled as events of interest using standard procedures (60, 61).

#### Statistical power calculation

This is a superiority RCT. Empirical power analysis was performed based on preliminary data from our pilot study and designed to ensure sufficient power to detect a clinically significant moderation effect in percent weight loss at month eight (i.e., the difference of differences between individuals who are characterized as habitual vs. not habitual HFD consumers at baseline using the DFS). A simulation using linear mixed effect models with linear contrasts using t statistics was run for 1,000 times. If we divide participants into HFD and low-fat diet (LFD) consumers using a median split, with a sample size of 100 people with 20% attrition, we achieve 90% power to detect a modulatory effect of 7% more weight loss in the HFD stratum compared to that in LFD stratum with an alpha level at 0.05, two-tailed. If we estimate 13% attrition for the secondary endpoint at month 16, which is reasonable since study demands are very low for the final 8 months, we will end with 70 participants, which results in 83% power to detect 7% more weight loss from baseline.

A second consideration is ensuring sufficient power for the neuroimaging analysis. We test if striatal and insular response to milkshake changes in the OEA vs. Placebo groups from baseline to the primary endpoint (month eight) using baseline DFS score as a covariate. Averaging across prospective effects from previous studies using a similar paradigm, striatal response to milkshake showed a large correlation with change in BMI (60–63). Applying standard power estimation procedures based on *N* = 80 at the primary endpoint and assuming a two-tailed alpha of 0.05, we will have a power of greater than 80% to detect the same magnitude of time by group interaction using a mixed effects regression analysis. We also attempt to detect a neural fingerprint that predicts weight loss on OEA based on whole-brain connectivity-based predictive maps (CPM). CPM is a newly developed data-driven predictive modeling technique, and a sample size of 50 has been recommended (64).

#### Analysis for aim 1

To test the hypothesis that fat intake will strongly moderate the ability of OEA to produce clinically significant weight loss maintenance four- and 12-months after LEARN^®^, a mixed effect model will be used with treatment (OEA vs. Placebo) and time as main effects and fat intake (LFD VS HFD) as the moderator variable. The three-way interaction will be used to examine the moderation effect. We will use an unstructured covariance matrix to account for the potential correlation of repeated measures within the same individual. This approach is tolerant to missing data by using all available data and maximum likelihood estimation method (65) SAS 9.4 (NC, Cary) will be used for the analysis. Data will be presented and visualized using a line or scatter plot.

#### Analysis for aim 2

The goal of aim 2 is to test if the DFS is associated with measures of saturated fat intake and to optimize a clinically useful stratification strategy. First, to test if the DFS is associated with measures of saturated fat intake, the fat score from the DFS will be designated as the independent variable in regression models with the saturated fat intake from the ASA24 and 3-day food diary as the dependent variables. If the DFS fat score is a valid reflection of saturated fat intake, then the DFS will account for significant variance in the ASA24 and 3-day saturated fat intake.

To test the prediction that baseline striatal response to milkshake predicts weight loss in the OEA but not the placebo group, we will analyze the neuroimaging data from the first full assessment and regress percent weight loss from baseline to the primary endpoint (month eight). First, we will use a custom pipeline to preprocess the data, before running statistical analyses using tools from the FMRIB Software Library (FSL version 5.09^2^). Non-brain tissues (e.g., scalp and CSF) will be removed using an automated brain extraction tool (66). Time series will be realigned to correct for small head movements using MCFLIRT (67). The susceptibility-induced distortions will then be estimated based on the images with reversed phase-encoding using FSL’s TOPUP tool (68) and applied for distortion correction of the functional images.

Next, we will submit the baseline data to post-processing using SPM12 software in MATLAB R2016b. Design matrices identifying the onset and duration of each event will be created for each participant. MS and Tlss presentations are modeled as events of interest using our standard procedures (60, 61). An ANOVA contrasting brain response to MS-Tlss at baseline with OEA or placebo treatment as the between-subjects variable. Next, we will regress percent weight change from baseline to the primary endpoint for each group and compare the regression contrast maps. A mask of the striatum will be extracted from the Oxford-GSK- Imanova structural striatal atlas (69) and responses will be considered significant if *p* < 0.05 corrected for multiple comparisons using the family-wise error rate (FWE) across voxels in the mask. Unpredicted peaks will be considered significant at *p* < 0.05 corrected for multiple comparisons using FWE across the whole brain. If our prediction is correct, then the contrast regression map will reveal significant differential response in the striatum in the OEA vs. the placebo group. As a secondary analysis, we will also test for differential responses as a function of group and time in the insular cortex. We will use a functional mask from a study identifying brain regions where response to MS changes as a function of daily consumption of a high fat/sugar snack for 8 weeks (Edwin et al., in preparation). This includes the left and right insula and overlying operculum. The neuroimaging data will be presented as statistical parametric maps of significantly activated voxels overlayed on an average anatomical image.

To test the prediction that a “neural fingerprint” can be isolated to predict weight loss on OEA, CPMs will be generated from the entire fMRI time series, including MS and Tlss deliveries as well as inter-stimulus periods. We first use a whole-brain functional atlas (70) to parcellate the brains of each subject into 268 nodes that maximize the similarity of voxel- wise time courses within each node. 268 × 268 connectivity matrices for each run will then be subsequently created in BioImage Suite by averaging the blood-oxygen-level-dependent (BOLD) signal of each voxel within nodes (71). Connectivity matrices will be Fisher Transformed to convert the skewed distribution of Pearson’s r (or Spearman’s Rho) values to an approximately normal distribution. Matrices are then averaged across runs to create a mean matrix for each participant, which is then collapsed across participants to create 268 × 268 × N-subject matrices. In the N-1 training set, CPM uses a linear regression (i.e., Pearson’s r or Spearman’s Rho) to correlate each edge (i.e., connection) in each connectivity matrix to percent weight loss. Each edge, as a result, is then associated with a particular *p*-value and the most relevant edges are selected for further use *via* thresholding (*p* = 0.01). Edges that are positively correlated with percent weight loss constitute a “positive network,” while edges whose strengths are negatively correlated with percent weight loss constitute a “negative network.” Together, these positive and negative networks make up the combined network/model. Single-subject summary values are then calculated by separately summing the edge-strengths of positive and negative networks (72). These positive and negative “network strengths” are used in a linear model with behavior and applied prospectively to the left-out subjects network strengths to generate estimated behavioral scores. To determine the significance of the obtained correlations between observed and estimated behavioral scores, we will conduct 1,000 repetitions of CPM using randomly shuffled observed scores to generate predicted scores and networks. The 1,000 correlation coefficients obtained from permutation testing will be used to comprise a null distribution against which the correlation coefficients can be tested for significance. Positive and negative brain functional networks predicting weight loss will be presented for the OEA and Placebo groups.

To identify baseline measures (i.e., dietary, blood, metabolic, perceptual, cognitive, and brain) that best predict weight loss on OEA, LASSO regression will be used. For IC, the first 5-min will be discarded and the 5-min segment with the least fluctuations of CO_2_ and O_2_ values with a coefficient of variation ≤ 10% will be identified. The RER and REE will be averaged across the 5-min. To account for weight loss these values will be linearly adjusted for fat free mass (44). To determine the optimal set of measures to best predict outcome, LASSO regression models will be conducted. LASSO regression maximizes the amount of variance while minimizing predictor redundancy (36). Importantly, our goal is a clinically useful stratification strategy. Therefore, even if neural measures are the best predictors, stratification requiring neuroimaging is generally not practical. In this case we will perform principal component analyses to test for more clinically tractable predictors (e.g., DFS + inflammatory markers). Lists of significant predictors and line plots with outcome measurements will be presented.

#### Analysis for aim 3

The primary of goal of aim 3 is to test a model of OEA effectiveness (Figure 1), where HFD reduces fat sensing and signaling to the brain, which in turn shifts preference to higher fat foods thereby promoting HFD and further weight gain. Importantly, we predict that the gut-brain pathway blunting and its reversal, are associated with diet rather than weight change, suggesting that individuals with obesity who maintain an HFD might benefit from OEA supplementation, whereas those that do not consume an HFD may not. Data will be presented as line and/or scatter plots with participants stratified into LFD and HFD consumers.

To test the prediction that weight loss is associated with shifts in fat preference mediated by increases in striatal response to milkshake in the OEA but not placebo group we will perform mediation analyses. First, we will test for the presence of correlations between our 3 variables (percent change in fat concentration preference; percent change in body weight and change in striatal response to MS-Tlss post—baseline). We predict significant correlations in the OEA but not the placebo group. Using the MATLAB based mediation toolbox (73), a 3-variable path model will be specified in which the predictor is change in fat concentration preference, the mediator is the subject wise beta estimate of striatal response to MS-Tlss at post—baseline in OEA vs. Placebo and the outcome is% change in body weight. As a secondary analysis we will substitute striatal response with insular response, since changes in BOLD response to MS-Tlss in the insula are observed after HFD. Relations between weight loss outcomes, fat preference, and brain response will be presented using scatter plots and statistical parametric maps of significantly activated voxels overlayed on an average anatomical image.

In addition, we will conduct mediation analyses to examine whether the weight loss in the high-fat OEA group is mediated by reduction in fat intake. First, we will model self-reported monthly weight loss and test if the reductions in saturated fat intake measured monthly by the DFS and ASA24 mediate weight loss. The self-reported weight will be validated against weigh-in weights during the LEARN^®^, program during the first 4 months. A similar model will also be constructed using the body weight measured over the four time points (two full assessments and two short assessments). The mediation effect of fat preference and fat intake that are measured at same time will be tested in the model. We will also test our alternative predictions that cognitive measures, food reinforcement, REE and RER act as moderators for OEA effectiveness. Linear growth curve models will be used for these analyses. Effects of fat intake, cognitive measures, food reinforcement, REE, and weight loss outcomes will be presented using line, bar, and violin plots.

## Discussion

Current treatments for obesity (2) can produce short-term weight loss and associated health benefits (6) but weight-regain frequently follows and is associated with return of associated morbidity (7). The high rates of recidivism that characterize this complex chronic medical problem highlight the pressing need for identifying strategies to improve weight loss maintenance (2–5, 74). The current study takes a precision medicine approach aiming to improve outcomes for individuals who habitually consume a HFD. Specifically, we have designed DAWLY, an RCT to test if habitual fat intake moderates the effect of OEA on weight loss and weight loss maintenance in the context of a well-established longstanding evidence-based behavioral weight loss program (LEARN^®^) (58). Prior studies using supplementation with OEA to improve weight loss have been met with limited success (75). Since HFD depletes OEA and acute administration of OEA can reverse the effects of HFD on vagal afferent induction of DA release, we reasoned that OEA supplementation might be most beneficial for individuals who consume a HFD. Our primary aim is to test this hypothesis. In addition, we propose to test the validity of the DFS by correlating questionnaire responses with other measures of fat intake and to optimize a clinically useful stratification strategy, to define measures that can be used to identify individuals who will most benefit from OEA supplementation. Finally, we will test a proposed model of OEA effectiveness (Figure 1).

There is mounting evidence for the role of the brain in nutrient sensing and food choice in developing obesity (76). Preclinical work has elucidated a novel gut-brain pathway that regulates striatal DA release during lipid ingestion to support the reinforcing value, preference, and intake of fat. Critically, this pathway is blunted by HFD in the absence of weight gain, resulting in reduced preference for low-fat foods and severe blunting of DA response to the gastric infusion of lipids in mice. The blunted DA response is rescued by OEA administration to the HFD fed animals and this is accompanied by increased preference toward lower fat emulsions (27, 11). This leads to a model where HFD reduces fat sensing and signaling to the brain, which in turn shifts preference to higher fat foods promoting HFD and further weight gain (Figure 1). Importantly, the pathway blunting and its reversal, are associated with diet rather than weight gain, suggesting that individuals with obesity who maintain an HFD might benefit from OEA supplementation, whereas LFD-consumers may not. Accordingly, OEA has been demonstrated to aid in weight loss in rodents made obese by a HFD (25, 27). However, in humans, studies that do not take diet into account report more modest effects (28, 29). The preclinical work suggests that better outcomes may be obtained if treatment is targeted to HFD consumers.

We will therefore evaluate the efficacy of OEA to shift fat preference toward lower fat food, decrease fat intake and increase activation of central DA circuits to facilitate weight loss and weight loss maintenance in individuals with OW/OB undergoing the LEARN^®^ behavioral weight loss intervention. In addition, the neuropsychological and food reinforcement measures will allow us to examine other DA-dependent functions that may be influenced by HFD and/or mediate OEA effectiveness. There is strong evidence that HFD can alter other DA-dependent cognitive function (38), many of which are known risk factors for weight gain. Therefore, it is possible that HFD-induced blunting of the reinforcing vagal afferent pathway and its reversal by OEA influences other DA-dependent behaviors, which also contribute to OEA supplementation efficacy. Accordingly, in rodents, OEA administration decreases cue-induced reinstatement of alcohol consumption, suggesting that it can affect reward circuits relevant for cue-induced feeding (77). In humans, striatal response to milkshake is associated with false alarm rate of impulsivity in individuals with OW/OB (60) and plasma OEA levels are associated with brain response to food-related stimuli in limbic regions important for reinforcement (78).

Finally, REE and RER will be determined using IC. OEA has been shown to stimulate lipolysis (79) and increase lipid oxidation, reflected in lower RER in mice, an effect dependent upon PPARa (peroxisome proliferator-activated receptor alpha)-signaling (80). Discovering mediators of OEA effectiveness is critical for understanding mechanisms and identifying potential targets for supplemental intervention.

A potential limitation in the current study is the challenges in recruitment and retention of participants during the trial. Subjects with OW/OB are likely to have obesity-related comorbidities such as hypertension and type-2 diabetes. Otherwise, healthy individuals are recruited through flyers and advertisements on billboards, radio, newspaper, and social media. We have a number of strategies in place to maximize retention and minimize attrition, including flexibility with session schedules, maintaining monthly contact with participants, and incentives.

In summary, existing treatments for obesity have a high rate of recidivism (2). Focusing on interventions that target neural circuits regulating food choice is supported by mounting evidence for the role of the brain in nutrient sensing and food choice in developing obesity (9). Preclinical work has elucidated a novel vagal afferent pathway critical for sensing dietary lipids regulating the reinforcing value of fat is blunted by a HFD, which is reversed upon OEA infusion with concomitant shifts to greater preference for low-fat foods and rescued blunting of DA response to the gastric infusion of lipids (10, 11). The current translational study focuses specifically on weight loss maintenance. This study will optimize a stratification strategy, using neural, metabolic and behavioral measures to identify individuals who will maintain clinically significant weight loss by daily OEA supplementation following a gold-standard behavioral weight loss program. If successful, the study will support a novel gut-brain target for weight loss maintenance in combination with a precision medicine approach to behavioral weight loss plus supplementation that is easy and inexpensive to implement.

## Ethics statement

The studies involving human participants were reviewed and approved by Yale Human Investigations Committee. The patients/participants provided their written informed consent to participate in this study.

## Author contributions

DS designed the study. MW and CG provided expertise, oversight, and training for the behavioral weight-loss trial. KF provided expertise and training on all nutritional measurements and analyses. FL performed the power analyses and provided expertise on the statistical analysis plan. XF and DS drafted the manuscript. All authors revised and approved the final version of the manuscript.

## Footnotes

1
http://www.caymanchem.com

2
www.fmrib.ox.ac.uk/fsl

## References

[1] Hales CM Fryar CD Ogden CL. *Prevalence of Obesity and Severe Obesity Among Adults: United States, 2017–2018.* Hyattsville, MD: National Center for Health Statistics (2020).

[2] Wadden TA Tronieri JS Butryn ML. Lifestyle modification approaches for the treatment of obesity in adults. *Am Psychol.* (2020) 75:235–51. 10.1037/amp0000517 32052997 PMC7027681

[3] Wing RR Phelan S. Long-term weight loss maintenance. *Am J Clin Nutr.* (2005) 82:222S–5S. 10.1093/ajcn/82.1.222S 16002825

[4] Franz MJ Vanwormer JJ Crain AL Boucher JL Histon T Caplan W Weight-loss outcomes: a systematic review and meta-analysis of weight-loss clinical trials with a minimum 1-year follow-up. *J Am Diet Assoc.* (2007) 107:1755–67. 10.1016/j.jada.2007.07.017 17904936

[5] Sawamoto R Nozaki T Nishihara T Furukawa T Hata T Komaki G Predictors of successful long-term weight loss maintenance: a two-year follow-up. *Biopsychosoc Med.* (2017) 11:14. 10.1186/s13030-017-0099-3 28592990 PMC5460352

[6] Wing RR Bray GA Cassidy-Begay M Clark JM Coday M Look Ahead Research Group Effects of intensive lifestyle intervention on all-cause mortality in older adults with type 2 diabetes and overweight/obesity: results from the look AHEAD study. *Diabetes Care.* (2022) 45:1252–9. 10.2337/figshare.19196288.v1 PMC9174966 35312758

[7] Wing RR Neiberg RH Bahnson JL Clark JM Espeland MA Hill JO Weight change during the postintervention follow-up of look AHEAD. *Diabetes Care.* (2022) 45:1306–14. 10.2337/dc21-1990 PMC9277114 35421225

[8] O’rahilly S Farooqi IS. Human obesity: a heritable neurobehavioral disorder that is highly sensitive to environmental conditions. *Diabetes.* (2008) 57:2905–10. 10.2337/db08-0210 18971438 PMC2570383

[9] Rossi MA Stuber GD. Overlapping brain circuits for homeostatic and hedonic feeding. *Cell Metab.* (2018) 27:42–56. 10.1016/j.cmet.2017.09.021 29107504 PMC5762260

[10] Han W Tellez LA Perkins MH Perez IO Qu T Ferreira J A Neural circuit for gut-induced reward. *Cell.* (2018) 175:665–678e623. 10.1016/j.cell.2018.08.049 30245012 PMC6195474

[11] Tellez LA Medina S Han W Ferreira JG Licona-Limon P Ren X A gut lipid messenger links excess dietary fat to dopamine deficiency. *Science.* (2013) 341:800–2. 10.1126/science.1239275 23950538

[12] Johnson PM Kenny PJ. Dopamine D2 receptors in addiction-like reward dysfunction and compulsive eating in obese rats. *Nat Neurosci.* (2010) 13:635–41. 10.1038/nn.2519 20348917 PMC2947358

[13] Mazzone CM Liang-Guallpa J Li C Wolcott NS Boone MH Southern M High-fat food biases hypothalamic and mesolimbic expression of consummatory drives. *Nat Neurosci.* (2020) 23, 1253–1266. 10.1038/s41593-020-0684-9 32747789 PMC7529959

[14] Adams WK Sussman JL Kaur S D’souza AM Kieffer TJ Winstanley CA. Long-term, calorie-restricted intake of a high-fat diet in rats reduces impulse control and ventral striatal D2 receptor signalling – two markers of addiction vulnerability. *Eur J Neurosci.* (2015) 42:3095–104. 10.1111/ejn.13117 26527415

[15] Hryhorczuk C Sharma S Fulton SE. Metabolic disturbances connecting obesity and depression. *Front Neurosci.* (2013) 7:177. 10.3389/fnins.2013.00177 24109426 PMC3791387

[16] Hryhorczuk C Florea M Rodaros D Poirier I Daneault C Des Rosiers C Dampened mesolimbic dopamine function and signaling by saturated but not monounsaturated dietary lipids. *Neuropsychopharmacology.* (2016) 41:811–21. 10.1038/npp.2015.207 26171719 PMC4707827

[17] Diep TA Madsen AN Holst B Kristiansen MM Wellner N Hansen SH Dietary fat decreases intestinal levels of the anorectic lipids through a fat sensor. *FASEB J.* (2011) 25:765–74. 10.1096/fj.10-166595 20959516

[18] Dipatrizio NV Piomelli D. Intestinal lipid-derived signals that sense dietary fat. *J Clin Invest.* (2015) 125:891–8. 10.1172/JCI76302 25642767 PMC4362267

[19] Laleh P Yaser K Abolfazl B Shahriar A Mohammad AJ Nazila F Oleoylethanolamide increases the expression of PPAR-alpha and reduces appetite and body weight in obese people: a clinical trial. *Appetite.* (2018) 128:44–9. 10.1016/j.appet.2018.05.129 29787831

[20] Tutunchi H Ostadrahimi A Saghafi-Asl M Roshanravan N Shakeri-Bavil A Asghari-Jafarabadi M Expression of NF-kappaB, IL-6, and IL-10 genes, body composition, and hepatic fibrosis in obese patients with NAFLD-Combined effects of oleoylethanolamide supplementation and calorie restriction: a triple-blind randomized controlled clinical trial. *J Cell Physiol.* (2021) 236:417–26. 10.1002/jcp.29870 32572955

[21] Kazemi M Lalooha F Nooshabadi MR Haghighian HK. Decreased dysmenorrhea pain in girls by reducing oxidative stress and inflammatory biomarkers following supplementation with oleoylethanolamide: a randomized controlled trial. *J Obstet Gynaecol Res.* (2022) 48:1212–21. 10.1111/jog.15196 35293068

[22] Fan A Wu X Wu H Li L Huang R Zhu Y Atheroprotective effect of oleoylethanolamide (OEA) targeting oxidized LDL. *PLoS One.* (2014) 9:e85337. 10.1371/journal.pone.0085337 24465540 PMC3896367

[23] Yang L Guo H Li Y Meng X Yan L Dan Z Oleoylethanolamide exerts anti-inflammatory effects on LPS-induced THP-1 cells by enhancing PPARalpha signaling and inhibiting the NF-kappaB and ERK1/2/AP-1/STAT3 pathways. *Sci Rep.* (2016) 6:34611. 10.1038/srep34611 27721381 PMC5056375

[24] Tutunchi H Naeini F Saghafi-Asl M Farrin N Monshikarimi A Ostadrahimi A. Effects of oleoylethanolamide supplementation on atherogenic indices and hematological parameters in patients with nonalcoholic fatty liver disease: a clinical trial. *Health Promot Perspect.* (2020) 10:373–82. 10.34172/hpp.2020.56 33312933 PMC7722997

[25] Rodriguez De Fonseca F Navarro M Gomez R Escuredo L Nava F Fu J An anorexic lipid mediator regulated by feeding. *Nature.* (2001) 414:209–12. 10.1038/35102582 11700558

[26] Begg DP Woods SC. Hedonic and homeostatic overlap following fat ingestion. *Cell Metab.* (2013) 18:459–60. 10.1016/j.cmet.2013.09.012 24093671 PMC3869225

[27] Fu J Oveisi F Gaetani S Lin E Piomelli D. Oleoylethanolamide, an endogenous PPAR-alpha agonist, lowers body weight and hyperlipidemia in obese rats. *Neuropharmacology.* (2005) 48:1147–53. 10.1016/j.neuropharm.2005.02.013 15910890

[28] Rondanelli M Opizzi A Solerte SB Trotti R Klersy C Cazzola R. Administration of a dietary supplement (N-oleyl-phosphatidylethanolamine and epigallocatechin-3-gallate formula) enhances compliance with diet in healthy overweight subjects: a randomized controlled trial. *Br J Nutr.* (2009) 101:457–64. 10.1017/S0007114508024008 18590587

[29] Mangine GT Gonzalez AM Wells AJ Mccormack WP Fragala MS Stout JR The effect of a dietary supplement (N-oleyl-phosphatidyl-ethanolamine and epigallocatechin gallate) on dietary compliance and body fat loss in adults who are overweight: a double-blind, randomized control trial. *Lipids Health Dis.* (2012) 11:127. 10.1186/1476-511X-11-127 23033919 PMC3490828

[30] Dumas JA Bunn JY Nickerson J Crain KI Ebenstein DB Tarleton EK Dietary saturated fat and monounsaturated fat have reversible effects on brain function and the secretion of pro-inflammatory cytokines in young women. *Metabolism.* (2016) 65:1582–8. 10.1016/j.metabol.2016.08.003 27621193 PMC5023067

[31] Gilbert JR Burger KS. Neuroadaptive processes associated with palatable food intake: present data and future directions. *Curr Opin Behav Sci.* (2016) 9:91–6. 10.1016/j.cobeha.2016.02.033

[32] Dalenberg JR Patel BP Denis R Veldhuizen MG Nakamura Y Vinke PC Short-term consumption of sucralose with, but not without, carbohydrate impairs neural and metabolic sensitivity to sugar in humans. *Cell Metab.* (2020) 31:493–502e497. 10.1016/j.cmet.2020.01.014 32130881 PMC7784207

[33] Thompson FE Kirkpatrick SI Subar AF Reedy J Schap TE Wilson MM The national cancer institute’s dietary assessment primer: a resource for diet research. *J Acad Nutr Diet.* (2015) 115:1986–95. 10.1016/j.jand.2015.08.016 26422452 PMC4663113

[34] Francis H Stevenson R. Validity and test-retest reliability of a short dietary questionnaire to assess intake of saturated fat and free sugars: a preliminary study. *J Hum Nutr Diet.* (2013) 26:234–42. 10.1111/jhn.12008 23190372

[35] Ortega RM Perez-Rodrigo C Lopez-Sobaler AM. Dietary assessment methods: dietary records. *Nutr Hosp.* (2015) 31(Suppl. 3):38–45. 25719769 10.3305/nh.2015.31.sup3.8749

[36] Hovens IB Dalenberg JR Small DM. A brief neuropsychological battery for measuring cognitive functions associated with obesity. *Obesity (Silver Spring).* (2019) 27:1988–96. 10.1002/oby.22644 31654505 PMC6868337

[37] D’ardenne K Savage CR Small D Vainik U Stoeckel LE. Core neuropsychological measures for obesity and diabetes trials: initial report. *Front Psychol* (2020) 11:554127. 10.3389/fpsyg.2020.554127 33117225 PMC7557362

[38] Small DM. Dopamine adaptations as a common pathway for neurocognitive impairment in diabetes and obesity: a neuropsychological perspective. *Front Neurosci.* (2017) 11:134. 10.3389/fnins.2017.00134 28400713 PMC5368264

[39] Sun X Luquet S Small DM. DRD2: bridging the genome and ingestive behavior. *Trends Cogn Sci.* (2017) 21:372–84. 10.1016/j.tics.2017.03.004 28372879 PMC5745142

[40] Kehagia AA Barker RA Robbins TW. Cognitive impairment in Parkinson’s disease: the dual syndrome hypothesis. *Neurodegener Dis.* (2013) 11:79–92. 10.1159/000341998 23038420 PMC5079071

[41] Lange KW Robbins TW Marsden CD James M Owen AM Paul GM. L-dopa withdrawal in Parkinson’s disease selectively impairs cognitive performance in tests sensitive to frontal lobe dysfunction. *Psychopharmacology (Berl).* (1992) 107:394–404. 10.1007/BF02245167 1615139

[42] Elliott R Sahakian BJ Matthews K Bannerjea A Rimmer J Robbins TW. Effects of methylphenidate on spatial working memory and planning in healthy young adults. *Psychopharmacology (Berl).* (1997) 131:196–206. 10.1007/s002130050284 9201809

[43] Waltz JA Frank MJ Wiecki TV Gold JM. Altered probabilistic learning and response biases in schizophrenia: behavioral evidence and neurocomputational modeling. *Neuropsychology.* (2011) 25:86–97. 10.1037/a0020882 21090899 PMC3050485

[44] Compher C Frankenfield D Keim N Roth-Yousey L Evidence Analysis Working Group. Best practice methods to apply to measurement of resting metabolic rate in adults: a systematic review. *J Am Diet Assoc.* (2006) 106:881–903. 10.1016/j.jada.2006.02.009 16720129

[45] Becker GM Degroot MH Marschak J. Measuring utility by a single-response sequential method. *Behav Sci.* (1964) 9:226–32. 10.1002/bs.3830090304 5888778

[46] Perszyk EE Hutelin Z Trinh J Kanyamibwa A Fromm S Davis XS Fat and carbohydrate interact to potentiate food reward in healthy weight but not in overweight or obesity. *Nutrients.* (2021) 13:1203. 10.3390/nu13041203 33917347 PMC8067354

[47] Difeliceantonio AG Coppin G Rigoux L Edwin Thanarajah S Dagher A Tittgemeyer M Supra-additive effects of combining fat and carbohydrate on food reward. *Cell Metab.* (2018) 28:,33–44e33. 10.1016/j.cmet.2018.05.018 29909968

[48] Mason AE Vainik U Acree M Tomiyama AJ Dagher A Epel ES Improving assessment of the spectrum of reward-related eating: the RED-13. *Front Psychol.* (2017) 8:795. 10.3389/fpsyg.2017.00795 28611698 PMC5447741

[49] Mennella JA Finkbeiner S Reed DR. The proof is in the pudding: children prefer lower fat but higher sugar than do mothers. *Int J Obes (Lond).* (2012) 36:1285–91. 10.1038/ijo.2012.51 22546773 PMC3429629

[50] Green BG Dalton P Cowart B Shaffer G Rankin K Higgins J. Evaluating the ‘labeled magnitude scale’ for measuring sensations of taste and smell. *Chem Senses.* (1996) 21:323–34. 10.1093/chemse/21.3.323 8670711

[51] Lim J Wood A Green BG. Derivation and evaluation of a labeled hedonic scale. *Chem Senses.* (2009) 34:739–51. 10.1093/chemse/bjp054 19833660 PMC2762053

[52] Beck AT Steer RA Ball R Ranieri W. Comparison of Beck depression inventories -IA and -II in psychiatric outpatients. *J Pers Assess.* (1996) 67:588–97. 10.1207/s15327752jpa6703_13 8991972

[53] Craig CL Marshall AL Sjostrom M Bauman AE Booth ML Ainsworth BE International physical activity questionnaire: 12-country reliability and validity. *Med Sci Sports Exerc.* (2003) 35:1381–95. 10.1249/01.MSS.0000078924.61453.FB 12900694

[54] Tutunchi H Ostadrahimi A Saghafi-Asl M Hosseinzadeh-Attar MJ Shakeri A Asghari-Jafarabadi M Oleoylethanolamide supplementation in obese patients newly diagnosed with non-alcoholic fatty liver disease: effects on metabolic parameters, anthropometric indices, and expression of PPAR-alpha, UCP1, and UCP2 genes. *Pharmacol Res.* (2020) 156:104770. 10.1016/j.phrs.2020.104770 32217148

[55] Oveisi F Gaetani S Eng KT Piomelli D. Oleoylethanolamide inhibits food intake in free-feeding rats after oral administration. *Pharmacol Res.* (2004) 49:461–6. 10.1016/j.phrs.2003.12.006 14998556

[56] Thabuis C Destaillats F Lambert DM Muccioli GG Maillot M Harach T Lipid transport function is the main target of oral oleoylethanolamide to reduce adiposity in high-fat-fed mice. *J Lipid Res.* (2011) 52:1373–82. 10.1194/jlr.M013391 21515921 PMC3111743

[57] Brownell KD. *LEARN Program for Weight Management 2000.* Dallas, TX: American Health Publishing Company (2000).

[58] Wadden TA Berkowitz RI Womble LG Sarwer DB Phelan S Cato RK Randomized trial of lifestyle modification and pharmacotherapy for obesity. *N Engl J Med.* (2005) 353:2111–20. 10.1056/NEJMoa050156 16291981

[59] Grilo CM White MA Masheb RM Ivezaj V Morgan PT Gueorguieva R. Randomized controlled trial testing the effectiveness of adaptive “SMART” stepped-care treatment for adults with binge-eating disorder comorbid with obesity. *Am Psychol.* (2020) 75:204–18. 10.1037/amp0000534 32052995 PMC7027689

[60] Babbs RK Sun X Felsted J Chouinard-Decorte F Veldhuizen MG Small DM. Decreased caudate response to milkshake is associated with higher body mass index and greater impulsivity. *Physiol Behav.* (2013) 121:103–11. 10.1016/j.physbeh.2013.03.025 23562867 PMC3731396

[61] Sun X Kroemer NB Veldhuizen MG Babbs AE De Araujo IE Gitelman DR Basolateral amygdala response to food cues in the absence of hunger is associated with weight gain susceptibility. *J Neurosci.* (2015) 35:7964–76. 10.1523/JNEUROSCI.3884-14.2015 25995480 PMC4438134

[62] Stice E Spoor S Bohon C Small DM. Relation between obesity and blunted striatal response to food is moderated by TaqIA A1 allele. *Science.* (2008) 322:449–52. 10.1126/science.1161550 18927395 PMC2681095

[63] Geha PY Aschenbrenner K Felsted J O’malley SS Small DM. Altered hypothalamic response to food in smokers. *Am J Clin Nutr.* (2013) 97:15–22. 10.3945/ajcn.112.043307 23235196 PMC3522134

[64] Scheinost D Noble S Horien C Greene AS Lake EM Salehi M Ten simple rules for predictive modeling of individual differences in neuroimaging. *Neuroimage.* (2019) 193:35–45. 10.1016/j.neuroimage.2019.02.057 30831310 PMC6521850

[65] Allison PD. *Handling Missing Data by Maximum Likelihood.* Haverford, PA: Statistical Horizons (2012).

[66] Smith SM. Fast robust automated brain extraction. *Hum Brain Mapp.* (2002) 17:143–55. 10.1002/hbm.10062 12391568 PMC6871816

[67] Jenkinson M Bannister P Brady M Smith S. Improved optimization for the robust and accurate linear registration and motion correction of brain images. *Neuroimage.* (2002) 17:825–41. 10.1016/s1053-8119(02)91132-8 12377157

[68] Smith SM Jenkinson M Woolrich MW Beckmann CF Behrens TE Johansen-Berg H Advances in functional and structural MR image analysis and implementation as FSL. *Neuroimage.* (2004) 23(Suppl. 1):S208–19. 10.1016/j.neuroimage.2004.07.051 15501092

[69] Tzourio-Mazoyer N Landeau B Papathanassiou D Crivello F Etard O Delcroix N Automated anatomical labeling of activations in SPM using a macroscopic anatomical parcellation of the MNI MRI single-subject brain. *Neuroimage.* (2002) 15:273–89. 11771995 10.1006/nimg.2001.0978

[70] Shen X Tokoglu F Papademetris X Constable RT. Groupwise whole-brain parcellation from resting-state fMRI data for network node identification. *Neuroimage.* (2013) 82:403–15. 10.1016/j.neuroimage.2013.05.081 23747961 PMC3759540

[71] Joshi A Scheinost D Okuda H Belhachemi D Murphy I Staib LH Unified framework for development, deployment and robust testing of neuroimaging algorithms. *Neuroinformatics.* (2011) 9:69–84. 10.1007/s12021-010-9092-8 21249532 PMC3066099

[72] Shen X Finn ES Scheinost D Rosenberg MD Chun MM Papademetris X Using connectome-based predictive modeling to predict individual behavior from brain connectivity. *Nat Protoc.* (2017) 12:506–18. 28182017 10.1038/nprot.2016.178 PMC5526681

[73] Wager TD Davidson ML Hughes BL Lindquist MA Ochsner KN. Prefrontal-subcortical pathways mediating successful emotion regulation. *Neuron.* (2008) 59:1037–50. 10.1016/j.neuron.2008.09.006 18817740 PMC2742320

[74] Leblanc ES Patnode CD Webber EM Redmond N Rushkin M O’connor EA. Behavioral and pharmacotherapy weight loss interventions to prevent obesity-related morbidity and mortality in adults: updated evidence report and systematic review for the US preventive services task force. *JAMA.* (2018) 320:1172–91. 30326501 10.1001/jama.2018.7777 PMC13151892

[75] Cazzola R Rondanelli M. N-Oleoyl-phosphatidyl-ethanolamine and epigallo catechin-3-gallate mitigate oxidative stress in overweight and class I obese people on a low-calorie diet. *J Med Food.* (2020) 23:319–25. 10.1089/jmf.2019.0145 31928490

[76] Lam TKT. Neuronal regulation of homeostasis by nutrient sensing. *Nat Med.* (2010) 16:392–5. 10.1038/nm0410-392 20376051

[77] Bilbao A Serrano A Cippitelli A Pavon FJ Giuffrida A Suarez J Role of the satiety factor oleoylethanolamide in alcoholism. *Addict Biol.* (2016) 21:859–72. 10.1111/adb.12276 26037332 PMC4668242

[78] Grosshans M Schwarz E Bumb JM Schaefer C Rohleder C Vollmert C Oleoylethanolamide and human neural responses to food stimuli in obesity. *JAMA Psychiatry.* (2014) 71:1254–61. 10.1001/jamapsychiatry.2014.1215 25229205

[79] Guzman M Lo Verme J Fu J Oveisi F Blazquez C Piomelli D. Oleoylethanolamide stimulates lipolysis by activating the nuclear receptor peroxisome proliferator-activated receptor alpha (PPAR-alpha). *J Biol Chem.* (2004) 279:27849–54. 10.1074/jbc.M404087200 15123613

[80] Caillon A Duszka K Wahli W Rohner-Jeanrenaud F Altirriba J. The OEA effect on food intake is independent from the presence of PPARalpha in the intestine and the nodose ganglion, while the impact of OEA on energy expenditure requires the presence of PPARalpha in mice. *Metabolism.* (2018) 87:13–7. 10.1016/j.metabol.2018.06.005 29936173

